# Cost of illness due to respiratory syncytial virus acute lower respiratory tract infection among infants hospitalized in Argentina

**DOI:** 10.1186/s12889-024-17878-3

**Published:** 2024-02-10

**Authors:** Julia Dvorkin, Emiliano Sosa, Elizabeth Vodicka, Ranju Baral, Andrea Sancilio, Karina Dueñas, Andrea Rodriguez, Carlos Rojas-Roque, Patricia B. Carruitero, Fernando P. Polack, Clint Pecenka, Romina Libster, Mauricio T. Caballero

**Affiliations:** 1 Fundación Infant, Gavilán 94, Ciudad Autónoma de Buenos Aires, Buenos Aires, Argentina; 2 Escuela de Bio y Nanotecnologías, Universidad Nacional de San Martín (UNSAM), San Martín, Provincia de Buenos Aires Argentina; 3 Consejo de Investigaciones Cientificas y Tecnicas (CONICET), Buenos Aires, Argentina; 4 Center for Vaccine Innovation and Access, PATH, Seattle, WA USA; 5 Servicio de Pediatría, Hospital Evita de Lanús, Lanús, Provincia de Buenos Aires Argentina; 6 Servicio de Pediatría, Hospital Evita Pueblo de Berazategui, Berazategui, Provincia de Buenos Aires Argentina; 7 Centre for Health Economics, University of York, Heslington, York UK; 8 Facultad de Ciencias Económicas, Universidad Nacional de La Plata, La Plata, Argentina

**Keywords:** Cost of illness, Direct medical cost, Respiratory syncytial virus, Acute lower respiratory tract infection

## Abstract

**Background:**

Information is scarce regarding the economic burden of respiratory syncytial virus (RSV) disease in low-resource settings. This study aimed to estimate the cost per episode of hospital admissions due to RSV severe disease in Argentina.

**Methods:**

This is a prospective cohort study that collected information regarding 256 infants under 12 months of age with acute lower respiratory tract infection (ALRTI) due to RSV in two public hospitals of Buenos Aires between 2014 and 2016. Information on healthcare resource use was collected from the patient’s report and its associated costs were estimated based on the financial database and account records of the hospitals. We estimated the total cost per hospitalization due to RSV using the health system perspective. The costs were estimated in US dollars as of December 2022 (1 US dollar = 170 Argentine pesos).

**Results:**

The mean costs per RSV hospitalization in infants was US$587.79 (95% confidence interval [CI] $535.24 – $640.33). The mean costs associated with pediatric intensive care unit (PICU) admission more than doubled from those at regular pediatric wards ($1,556.81 [95% CI $512.21 – $2,601.40] versus $556.53 [95% CI $514.59 – $598.48]).

**Conclusions:**

This study shows the direct economic impact of acute severe RSV infection on the public health system in Argentina. The estimates obtained from this study could be used to inform cost-effectiveness analyses of new preventive RSV interventions being developed.

**Supplementary Information:**

The online version contains supplementary material available at 10.1186/s12889-024-17878-3.

## Background

Acute lower respiratory tract infection (ALRTI) due to respiratory syncytial virus (RSV) is a main cause of hospitalizations and one of the most common causes of preventable post neonatal deaths in the world [1, 2]. Globally, it is responsible for over 33 million episodes of infection in children under five years old and is a leading disease diagnosed in hospital admissions with 3.2 million episodes per year in 2019 [1]. The disease burden is most prominent among the poorest and youngest children from low- and middle-income countries (LMICs) [3, 4]. Surprisingly, recent studies have shown that nearly 70% of the deaths in low-income economies countries occurred at home, outside any health facilities [1, 5–8].

RSV disease among infants takes on added importance when considering the economic burden of illness [9]. Some estimations suggest that RSV accounts for 1.2 million discounted disability adjusted life-years (DALYs) and is associated with an economic toll of approximately €4.82 billion per year globally considering inpatients and outpatients under 5 years of age [9]. Most of this burden (65%) comes from LMICs, with almost 55% of global costs stemming from ALRTIs that require hospitalizations [9]. In addition, infants hospitalized due to RSV might require more complex care which leads to an increase in individual costs for health services [10].

Palivizumab (a monoclonal antibody against RSV F protein) was the first approved intervention for the prevention of severe ALRTI in pediatric patients [11]. Nevertheless, access to this prophylactic intervention is restricted in most LMICs. Some reasons are the high cost per dose, the difficulties in the administration logistics of several doses to achieve an efficacy close to 55%, and the recommendation for use which is limited to a specific subgroup of infants at very high risk [11–13]. Nirsevimab, a monoclonal antibody with an extended half-life that allows administration as a single dose to cover the whole RSV season, was recently approved by the US Food and Drug Administration (FDA) for the prevention of RSV lower respiratory tract disease in neonates and infants, and in children up to 24 months of age who remain vulnerable to severe RSV disease through their second RSV season [14, 15]. FDA has recently approved for US a vaccine for use in women during the middle of the third trimester of pregnancy to protect their babies against RSV up to 6 months after birth [16]. Moreover, many other vaccines and immune therapeutics technologies are also under clinical development and evaluation for children [17, 18].

In the scenario of new preventive interventions ahead, estimating the cost of illness (COI) for ALRTI due to RSV in LMICs is critical for several reasons [17]. First, most of the severe cases and deaths occur in these countries, where urgent implementation of preventive interventions is needed to diminish the impact of RSV disease. Second, COI studies provide relevant information on the economic burden of RSV and, therefore, the savings that could be achieved in the absence of RSV. Finally, COI studies can provide a more comprehensive understanding of the disease, including economic impact. A recent systematic review of COI for RSV identified three out of 44 costing studies conducted in LMICs and only one in Argentina, from the perspective of a private healthcare insurance [19, 20].

A gap in understanding remains around the economic impact of RSV episodes on previously healthy term infants attended in public hospitals in LMICs. This information is of special interest for stakeholders and decision makers to contribute setting priorities and guiding an evidence-based selection among available preventive interventions in contexts where resources are scarce. Therefore, the aim of this study was to determine the direct medical costs associated with ALRTI due to severe RSV disease in hospitalized infants in urban low-resource settings in Argentina.

## Methods

### Study design

This study estimated the cost of RSV hospitalization from the perspective of the public healthcare system [21]. Therefore, only the direct medical costs associated with providing RSV hospitalization services were considered. This study followed the methodological considerations published before for COI studies [22, 23].

### Data source and study setting

This study used data from a multicenter prospective cohort at two public hospitals of the southern Province of Buenos Aires, Argentina. The cohort was initiated in May 2014 and the follow up finished in June 2022. The cohort’s database contains information on healthcare resource utilization, RSV diagnosis, and demographic information from patients experiencing ALRTI [11]. Regulatory staff audited all the information uploaded to REDCap platform weekly [24]. All the queries were solved monthly with local investigators.

Although all inhabitants of Argentina can receive universal healthcare from public hospitals, the national healthcare system is divided into the following sectors: public, social security, and private [25–27]. Taxes and national or provincial budgets fund the public sector. Except for high-tech tertiary and quaternary national hospitals, all public hospitals belong to the provinces or the cities. The social security sector is composed of many different sick funds managed by trade unions to give health coverage to workers and their core family members [25, 26]. Finally, the private sector brings coverage to nearly six million individuals, who enroll on a voluntary out-of-pocket payment basis [25].

### Population

Infants 12 months of age or below were enrolled in the COI study during their first episode of severe ALRTI between May and September of 2014 to 2016 (RSV season in Argentina) in pediatric departments of two public hospitals of the Province of Buenos Aires, Argentina [10]. Severe ALRTI was defined as the presence of at least one manifestation of lower respiratory tract infection sign (cough, nasal flaring, indrawing of the lower chest wall, subcostal retractions, stridor, rales, rhonchi, wheezing, crackles or crepitations, or observed apnea) plus hypoxemia (peripheral oxygen saturation of < 95% at room air) or tachypnea (≥ 70 breaths per minute from 0 to 59 days of age, and ≥ 60 breaths per minute at 60 days of age or older) [10, 28, 29]. A panel of 10 respiratory pathogens, including RSV, was identified through quantitative real-time polymerase chain reaction (qPCR) by the research laboratory independently of the hospital's diagnostic method [6, 10]. This ensured the inclusion of the study participants based on rigorous and standardized pathogen identification. Of the 470 patients admitted in these two hospitals in the initial cohort study, all RSV positive patients (*n* = 256) were included for this COI study.

### Outcome of the study

The outcome of the study was defined as the direct medical cost of hospitalizations due to acute severe RSV disease. All costs in this study were initially calculated in Argentinian pesos (ARS) at the time of data collection and subsequently converted to US dollars using the average exchange rate reported by the Central Bank of Argentina on that specific date.

### Instruments

For the collection of healthcare resources, a data collection instrument was developed in REDCap [24]. The instrument was used to retrieve data on healthcare utilization during the child’s course of treatment in the hospital. If any data gaps were identified during the secondary analysis, a medical record review was conducted for any enrolled children for whom data were not available through the main study dataset.

### Cost estimation

The cost estimation methodology applied was the bottom-up costing method. Healthcare resources were identified and quantified from the data collection instrument developed in REDCap, while the unit cost of each healthcare resource was taken from the financial database and account records from the two public hospitals.

Per episode cost of RSV hospital admission was disaggregated in the following components:

- *Laboratory tests*: tests performed for diagnosis and monitoring during hospitalization such as blood count, C reactive protein, erythrocyte sedimentation rate, blood gases and ionogram test, and blood and urine cultures.
- *Labor cost:* includes wages for all health professionals in the hospital and calculated from physician and nurse monthly salaries, considering a differential pay for on-call hours, intensive care units, and number of beds per physician / nurse.
- *Drugs*: medications commonly used for ALTRI treatment (i.e., albuterol, corticosteroids, and antibiotics).
- *Feeding*: food provided daily by the hospital for patient and caregiver.
- *Imaging diagnosis*: chest X-ray and computed tomography scan.
- *Supplies*: This includes oxygen delivery devices (e.g., nasal cannula, Venturi mask, flexible connectors for mechanical ventilation, and endotracheal tube), nasogastric tubes, syringes, gloves, and other disposable items used for patient care.
- *Oxygen supply*: cost per cubic meter of oxygen was taken from hospital administration and then multiplied by oxygen consumption per day (calculated according to the type of oxygen device and based on national recommendations) [30].
- *Overhead*: includes administration cost, maintenance cost, bedding and linens. The county paid for items such as heating, water, electricity, and lighting, which, therefore, were not considered hospital expenses. We estimated overhead costs based on an output allocation base, which looked at the cost as a proportion to the units of service outputs—in other words, the overall overhead cost for the total of hospital beds in each hospital. Afterward, we multiplied that amount by the proportion of beds occupied by the RSV ALRTI patients in each hospital.
- *Equipment depreciation*: includes equipment for mechanical ventilation (infusion pump, imaging, etc.). We estimated the equipment depreciation using a 10-year useful lifetime and a linear depreciation approach. Equipment acquisition costs were retrieved from hospital financial reports.

### Sample size and statistical analyses

Providing an estimated mean cost with adequate precision required a sufficient sample size. Precision was set at ± 10% of the mean cost, with coefficient of variation assumed to be 0.5, and *z*-score equal to 1.96. Sample size was calculated based on the annual number of RSV severe cases in each setting using the following formula [31]:

$$N={\left(\frac{{precision}^{2}}{{Coefficient \,of Variation}^{2}\times {Z}^{2}}+\frac{2}{Annual \,Cases}\right)}^{-1}$$

We aimed to enroll approximately 250 patients for assessing the costs of acute severe RSV disease.

We analyzed data using Stata (16.1, StataCorp LLC, College Station, TX). Descriptive statistics (frequency and percent) were used to assess demographics, clinical variables, and healthcare resource utilization. We applied the K-S test (Kolmogorov–Smirnov) to assess the normality of data. For normally distributed variables, we described data using means and standard deviations (SD); while we described data using the median and interquartile range for non-normal distributed variables. We described the cost of RSV hospitalization by using median and mean together with its 95% CI. We used the Wilcoxon signed rank test to compare the pediatric ward RSV hospitalization direct medical costs and the pediatric intensive care unit (PICU) RSV hospitalization direct medical costs.

### Ethical considerations

The institutional review boards at each participating hospital, the Province of Buenos Aires, and Vanderbilt University approved the study. Informed consent was obtained from all participating parents or guardians.

## Results

### Study population

This study included 256 infants 12 months of age and younger that were hospitalized due to severe RSV ALRTI. The study population was selected from low-income communities in a large regions located in the southern outskirts of the Buenos Aires metropolitan area [6, 10, 32, 33]. Most of the parents did not finish secondary school education (62.67%). Further, we found that 2.21% of the parents with incomplete education did not finish primary school education (three parents with illiteracy). The majority of the houses did not have a sewage system (60.87%), and several families lived in crowded conditions (28.52%) (Table 1) [33]. The minimum annual income needed to sustain a family of four members in Argentina in December 2022 was US$11,323 [34]. Eighty-two percent of the families did not achieve the minimum annual income needed to sustain a family.

**Table 1:** Characteristics of the study population

		**RSV-ALRTI (*N* = 256)**	**Ward (*n* = 248)**	**PICU (*n* = 8)**
**Socioeconomic**				
Tin or mud house, n/N (%)		13/256 (5.08)	13/248 (5.24)	0/8 (0.00)
No running water, n/N (%)		31/250 (12.40)	30/242 (12.40)	1/8 (12.50)
No sewage system, n/N (%)		154/253 (60.87)	150/245 (61.22)	4/8 (50.00)
Crowding (> 3 inhabitants/room), n/N (%)		73/256 (28.52)	71/248 (28.63)	2/8 (25.00)
Incomplete parental education, n/N (%)		136/217 (62.67)	133/210 (63.33)	3/7 (42.86)
Monthly family income in USD, mean (SD)		615.86 (359.95)	607.91 (356.95)	851.50 (403.31)
Median household inhabitants (IQR)		5 (4–6)	5 (4–6)	5 (4–6)
**Patient characteristics**				
Female, n/N (%)		114/256 (44.53)	110/248 (44.35)	4/8 (50)
Age in months (mean, SD)		5.14 (3.01)	5.20 (3.01)	3.22 (2.74)
Previous medical consultation, n/N (%)		101/256 (39.45)	99/248 (39.92)	2/8 (25)
Medical insurance, n/N (%)		13/249 (5.22)	13/241 (5.39)	0/8 (0)
Diagnosis on admission	Bronchiolitis, n/N (%)	188/256 (73.44)	181/248 (72.98)	7/8 (87.50)
	Pneumonia, n/N (%)	15/256 (5.86)	15/248 (6.05)	-
	Pertussis like syndrome, n/N (%)	8/256 (3.13)	8/248 (3.23)	-
	Previous wheezing, n/N (%)	9/256 (3.52)	9/248 (3.63)	-
	Another respiratory syndrome, n/N (%)	36/256 (14.06)	35/248 (14.11)	1/8 (12.50)^a^
Length of stay in days, median (IQR)		7 (4–10)	7 (4–9.5)	18.5 (10.5–22)
Oxygen requirement in days, median (IQR)		6 (3–8)	6 (3–8)	15 (6.5–20.5)

References: *RSV-ARLTI* respiratory syncytial virus acute lower respiratory tract infection, *SD* standard deviation, *IQR* interquartile range

^a^Sepsis

The mean age of hospitalization due to RSV disease was five months (SD 3.01). Overall, only eight of 256 patients (3.1%) were admitted to a PICU. The median length of hospital stay in the cohort was seven days (interquartile range [IQR] 4–10) and eleven days for patients admitted to the PICU (IQR 6.5–13.5). The length of hospitalization was significantly longer (*p* = 0.046) in infants under three months of age (median 8, IQR 4–11) and between three to six months old (median 8, IQR 4–10) compared with those older than six months of age (median 5, IQR 4–9). We found 9.4% of patients to have health complications (24/256), the most common of which was pneumonia (seen in 87.5% of the cases [21/24]).

### Use of healthcare resources

All care procedures were performed in the context of patient assistance. Nearly all the patients hospitalized due to RSV had at least one diagnostic and treatment procedure (Table 2). The most common diagnostic procedure in the cohort was immunofluorescence for viral diagnosis (98.8%), followed by chest X-ray (97.3%). Except for one patient who was hospitalized for a social condition, all participating infants required supplementary oxygen as supportive treatment. Nasal cannula was the most frequently used device, with only five infants requiring mechanical ventilation.

**Table 2:** Use and cost of diagnostic and treatment resources in the study population

	**RSV-ALRTI (*N* = 256)**				
	**Cost in USD per unit**	**Total**	**Ward**	**PICU**	**p**
		**(*n* = 256)**	**(*n* = 248)**	**(*n* = 8)**	
	$	n/N (%)	n/N (%)	n/N (%)	
**Diagnostic procedures**					
X-ray	12.00	249/256 (97.27)	241/248 (97.18)	8/8 (100)	1,000
Blood tests^a^	11.17	135/256 (52.53)	127/248 (51)	8/8 (100)	0.008
Blood cultures	15.00	54/256 (21.09)	46/248 (18.55)	8/8 (100)	0.000
RSV diagnostic test	3.50	253/256 (98.83)	245/248 (98.79)	8/8 (100)	1,000
**Drug utility**					
Analgesic/antipyretic^b^	0.38	76/256 (29.57)	74/248 (29.72)	2/8 (25)	1,000
Albuterol	5.00	243/256 (94.55)	215/248 (86.35)	7/8 (87.50)	1,000
Antibiotics^c^	4.11	65/256 (25.29)	59/248 (23.69)	6/8 (75)	0.004
Corticosteroids	1.27	46/256 (17.90)	42/248 (16.87)	4/8 (50)	0.036
**Supplementary oxygen**					
Nasal cannula	1.56	255/256 (99.61)	247/248 (99.60)	8/8 (100)	1,000
Venturi mask	1.30	49/256 (19.14)	43/248 (17.34)	6/8 (75)	0.001
Re-breather mask	1.56	12/256 (4.69)	9/248 (3.63)	3/8 (37.50)	0.004
Mechanical ventilation	6.06	5/256 (1.95)	0	5/8 (62.50)	0.000

*RSV-ALRTI* Respiratory syncytial virus acute lower respiratory tract infection, *PICU* Pediatric intensive care unit

^a^Blood count, erythrocyte sedimentation rate, blood gases, ionogram, glycemia, C-reactive protein

^b^Cost per dose for most used antipyretics (Ibuprofen, Dipyrone, Acetaminophen)

^c^Cost for one day of treatment for most used antibiotics (Amoxicillin, Ampicillin, Ampicillin-Sulbactam, Ceftriaxone, Clarithromycin, Clindamycin)

Although there is no evidence to support the regular administration of inhaled short-acting beta-2 adrenergic agonist (albuterol) for the treatment of hospitalized patients with viral bronchiolitis as a therapy for wheezing [17, 35], 95% of the participants received albuterol. Despite a very low rate of positive blood culture 4/42 (9.53%), antibiotics were prescribed in 25.3% of the patients, and ceftriaxone was the most frequently used (33.85% of those receiving antibiotics).

Overall, diagnostic procedures had higher unit costs compared to drugs and respiratory therapy supplies in the study (Table 2). Of these complementary methods, blood cultures presented the highest cost, followed by X-rays (not digitized), and by basic blood tests (including blood count, erythrocyte sedimentation rate, blood gases, blood ionogram, glycemia, and c-reactive protein).

### Direct medical costs of RSV hospitalizations

The total cost for 256 patients and 1,920 hospital bed days was US$150,474.58, and the mean direct medical cost for the RSV hospitalizations in two public hospitals of Buenos Aires (Table 3) was $587.79 (95% CI 535.25 – 640.33) per episode. The mean costs associated with PICU admission nearly tripled from those admissions at regular pediatric wards ($1,556.81 [95%CI 512.21 – 2601.40] versus $556.53 [95%CI 524.59 – 598.48]). Overall, the associated health workers labor cost was the main driver of total cost (Table 3), accounting for 65% of the hospital costs. Oxygen supply accounted for 8% and overhead costs for 15% of the total cost. Among patients admitted at the PICU, the laboratory testing, drugs, and oxygen supply accounted for similar proportions (6.1%, 6.9%, and 6.7%).

**Table 3:** Cost of acute RSV-ARLTI detailed by cost categories

Costs (mean, SD)	RSV-ARLTI (*N* = 256)			
	**Total (*n* = 256)**	**Ward (*n* = 248)**	**PICU (*n* = 8)**	**p**
**Laboratory test**				
Median	5.75	4.87	86.26	
IQR	3.50–15.02	3.50–10.25	53.49–128.76	0.000
Mean	13.24	9.17	93.85	
CI 95%	10.53–15.95	7.54–10.79	53.81–133.88	
**Labor costs**				
Median	358.23	307.06	562.94	
IQR	204.71–511.76	204.71–460.59	255.88–1731.18	0.106
Mean	383.15	363.39	995.51	
CI 95%	345.60–420.70	336.18–390.61	50.85–1940.17	
**Drugs**				
Median	5.36	5.36	116.40	
IQR	4.99–11.35	4.99–10.78	32.61–178.89	0.0013
Mean	12.71	9.68	106.68	
CI 95%	9.82–15.60	8.53–15.60	38.57–174.79	
**Feeding**^**a**^				
Median	12.60	12.60	67.83	
IQR	7.20–21.00	7.20–19.80	37.60–79.23	0.0001
Mean	22.26	20.96	62.38	
CI 95%	19.03–25.48	17.81–24.11	40.15–84.60	
**Imaging diagnosis**				
Median	12	12	36	
	12.00–12.00	12.00–12.00	12.00–36.00	0.000
Mean	12.44	11.80	32.5	
CI 95%	11.74–13.15	11.50–12.09	13.63–51.37	
**Supplies**				
Median	1.56	1.56	16.46	
IQR	1.56–3.12	1.56–3.12	8.58–18.02	0.001
Mean	4.60	4.32	13.30	
CI95%	3.90–5.30	3.64–5.00	7.18–19.42	
**Oxygen supply**				
Median	40.38	40.38	102.62	
IQR	20.19–60.56	20.19–59.72	54.68–144.68	0.0014
Mean	49.52	47.73	104.94	
CI95%	44.52–54.52	42.87–52.59	56.59–153.29	
**Overhead**^**b**^				
Median	85.4	73.20	128.10	
IQR	48.80–122.00	48.80–109.80	79.30–134.20	0.047
Mean	87.40	86.63	111.32	
CI95%	81.06–93.74	80.14–93.12	83.83–138.82	
**Equipment depreciation**^**c**^				
Median	1.1	1.1	34.99	
IQR	1.10–1.91	1.10–1.64	26.22–46.86	0.000
Mean	2.47	1.38	36.32	
CI95%	1.67–3.28	1.30–1.46	24.40–48.25	
**Total cost**				
Median	514.44	497.79	1293.31	
IQR	304.23–739.66	304.04–737.04	622.60–2386.02	0.0058
Mean	587.79	556.53	1556.81	
CI95%	535.25–640.33	514.59–598.48	512.21–2601.40	

*RVS-ALRTI* respiratory syncytial virus acute lower respiratory tract infection, *PICU* pediatric intensive care unit, *CI* Confidence interval

^a^Patient and one parent (either food or milk for patients under 6 months old)

^b^Bedding and linens, maintenance, and administrative expenses

^c^Includes equipment for mechanical ventilation, vital signs monitor, saturometer, infusion pump, and imaging

We did not find any differences in the total COI based on age groups; however, when we evaluated the costs by categories, we found that cost of laboratory tests and feeding were significantly higher in infants under three months of age (Supplementary Table 1). We did not have enough power to evaluate costs based on the presence of comorbidities by age groups.

Although individual measurement of emergency room (ER) care costs was not part of this study, we were able to estimate average costs based on information provided by hospitals. This estimate included the use of drugs, supplementary oxygen treatment for at least three hours, and healthcare personnel fees. Therefore, based on these data, we estimated that the basic care of a patient with ALRTI requiring oxygen prior to hospitalization was US$64.58.

### Annual economic burden due to RSV hospitalizations in Argentina

Based on Argentinean census information and the local incidence of severe RSV-ALRTI, we have estimated the annual economic burden of RSV hospitalization in infants younger than 12 months of age in country [1, 36]. For this approach we analyzed two possible scenarios—one with a lower and the other with higher burden of disease. In the lower burden of severe RSV disease, we estimated the national number of hospitalizations per year of 18,625 infants (incidence rate 0.27/1000 infants under 12 months of age) and a total cost of US$10,948,140 (95%CI 9,969,533 – 11,926,747). Under a higher burden scenario of 22,463 hospitalizations (incidence rate of 32.9/1000 infants), the total cost of RSV hospitalizations in Argentina were estimated to be $13,203,585 (95%CI 12,023,374—14,383,796) annually.

## Discussion

In this study, we have defined the severe RSV-ALRTI direct medical cost of infants 12 months of age or below hospitalized in two public institutions of Buenos Aires, Argentina. We showed that the medical cost of RSV hospitalizations has a substantial economic impact in Argentina. Published literature suggests that the direct medical costs per RSV hospital admission episode vary widely between countries and regions. Results from this study are comparable with previous estimates in the city of Buenos Aires, Argentina (US$529) [37], Bogotá, Colombia ($518) [38], and several cities in South Africa ($681) [39]. Nevertheless, the costs presented in this study diverge from those documented in both high-income and low-income countries [9, 40, 41]. Likewise, in 2015, Marcone and colleagues computed the cost of hospitalizations attributed to ALRTI caused by viral agents, including influenza, within a private hospital [37]. They reported a median cost of US$529, a figure remarkably close to the US$514 reported in this study [37]. Several meta-analyses assessing costs associated with pneumonia, whether attributable to various causes or specific to RSV or other respiratory viruses, have indicated broad ranges of costs on a global and regional scale. Nevertheless, the average cost per hospitalization episode typically hovers around US$500 for Latin American countries or nations with GDP per capita comparable to that of Argentina (US$ 13,650 in 2022). Consequently, it may be more accurate to compare and categorize ALRTI hospitalization expenses among countries based on their respective GDP per capita.

In relation to the costs by categories established by the study, we found that the greatest determinant of the COI was the cost of health personnel involved in the hospitalized patients’ medical care, for both ward (65% of the total cost) and PICU patients (64% of the mean total cost). This finding could be explained on the basis of specific measures regarding drugs acquisition by the state and low prices of diagnostic methods [42]. Since 2002, to increase access to pharmaceuticals for patients attending public health centers in the country, the Argentinean government has been implementing a program called "Remediar" [43]. This program involves state centralized acquisition of more than 40 essential multi-source medicines (in several presentations), which generates more than 75% of cost saving in commonly used medicines with respect to their market price [42].

Even though, Argentina is classified as an upper-middle income country by the World Bank [44], the study population had remarkably contrasted socioeconomic status in comparison with other parts of the country. We found that most of the study families lived under the poverty line based on the established Argentinean government thresholds [6, 32, 33, 45]. Ninety-five percent of the families did not have medical insurance and accessed public health care institutions mainly by public transportation [33]. Additionally, we found a soaring rate of incomplete parental education and high rate of poor living conditions.

This study had several limitations. First, the research focused on estimating the health costs related to hospitalized patients due to RSV-ALRTI and not to outpatients. We consider this an important issue for future studies since the greatest burden of RSV disease occurs at the outpatient level. Second, the social perspective was not considered as part of the study to avoid recall bias since economic burden was estimated retrospectively. Third, the overhead costs might be slightly underestimated in this study, mainly after the diversification of payments for services by various parties such as the municipality (local county administration), the ministry of health (regional state system), social security institutions, and other intermediaries. Fourth, the estimations of the annual economic burden due to RSV hospitalizations in the country were derived from only two public facilities located in Buenos Aires; therefore, inferences may not be representative to other regions in Argentina. Finally, this study has focused on determining the health costs only for the public health system; therefore, costs associated with RSV in the private health and social security systems might be significantly higher than in the public health system.

In conclusion, this study described in detail the hospital cost and economic burden of RSV-ALRTI in infants under 12 months of age in Buenos Aires, Argentina. The medical costs associated with severe ALRTI due to RSV reported in this study were substantial for the public health system of Argentina. Not surprisingly, the medical costs shown were comparable with previous reports from other middle-income countries [37–39, 46]. A detailed quantification of RSV-cost of illness is key for decision making, prioritizing future preventive interventions, and achieving effective allocation of health resources. Moreover, these data could be useful for estimating the full value of potentially avertable burden by vaccination [40]. Additionally, the information on cost categories reported in this study might enable policy makers and stakeholders to improve resource allocation strategies at health facilities. Further studies should explore the societal perspective, the outpatient economic burden of disease, and the impact of RSV in the country’s private sector.

### Supplementary Information

**Additional file 1: Supplementary Table 1.** Cost of acute RSV-ARLTI detailed by age groups.

## Data Availability

The datasets used and/or analyzed during the current study are available in Zenodo (https://zenodo.org/records/10440099) or from the corresponding author.

## Abbreviations

RSV: Respiratory syncytial virus
ALRTI: Acute lower respiratory tract infection
CI: Confidence interval
COI: Cost of illness
DALYs: Discounted disability adjusted life-years
ER: Emergency room
FDA: US Food and Drug Administration
IQR: Interquartile range
LMICs: Low- and middle-income countries
PICU: Pediatric intensive care unit
SD: Standard deviations
